# Diagnostic Accuracy of Urine Flow Cytometry (UF) in Urinary Tract Infection (UTI) Detection and Management: A Systematic Review and Meta-Analysis

**DOI:** 10.3390/diagnostics16091275

**Published:** 2026-04-23

**Authors:** Kai-Wei Chang, Chung-You Tsai, Shin-Mei Wong, Jeff Shih-Chieh Chueh, Shang-Jen Chang

**Affiliations:** 1 Department of Urology, National Taiwan University Hospital, Taipei City 100225, Taiwan; 2 Divisions of Urology, Department of Surgery, Far Eastern Memorial Hospital, New Taipei 220216, Taiwan; 3 Department of Electrical Engineering, Yuan Ze University, Taoyuan 320315, Taiwan

**Keywords:** urinary tract infection, flow cytometry, bacteriuria

## Abstract

**Background:** Urinary tract infections (UTIs) are prevalent bacterial infections associated with significant morbidity and healthcare burden. Traditional diagnosis relies on urine culture, which is limited by long turnaround times and potential contamination. Automated urine flow cytometry, particularly the UF-5000 (Sysmex Corporation, Kobe, Japan), offers rapid and precise screening through bacterial and white blood cell (WBC) quantification. This systematic review and meta-analysis evaluates the diagnostic accuracy of the UF-5000 for UTI screening. **Methods**: We searched PubMed, Embase, Science Direct, and Web of Science for studies assessing the UF-5000’s performance, including sensitivity and specificity, with a minimum sample size of 40 and at least 10 UTI cases. Quality assessment was performed using QUADAS-2. Pooled estimates for sensitivity, specificity, and agreement (kappa) were calculated using random-effects models. **Results:** Eighteen studies, encompassing 25,337 samples, were included in the analysis. Pooled sensitivity and specificity for bacterial count (nine studies) were 0.927 (95% CI, 0.872–0.959) and 0.751 (95% CI, 0.558–0.878), respectively. For WBC count (four studies), sensitivity was 0.897 (95% CI, 0.755–0.961) and specificity was 0.600 (95% CI, 0.293–0.844). The UF-5000 also demonstrated moderate agreement (pooled kappa 0.52, 95% CI, 0.08–0.79) in distinguishing Gram-negative bacteria. **Conclusions**: Despite heterogeneity across studies, the UF-5000 demonstrates high diagnostic accuracy, particularly high sensitivity, supporting its role as a useful UTI screening tool to rule out infection in clinical settings. The device further provides clinical value through its ability to assist in the differentiation of Gram-negative bacteria.

## 1. Introduction

As one of the most common bacterial infections globally, urinary tract infections (UTIs) are frequently encountered in clinical practice, with substantial morbidity and a considerable amount of healthcare burden. Although the etiology and epidemiology of UTIs have been well studied, how to rapidly and precisely make the diagnosis still remains a challenge [1]. UTI is defined as microbiological evidence of urinary infection (mostly a positive urine culture) along with related symptoms, such as fever, lower urinary tract symptoms (LUTS), or suprapubic tenderness. It can be further categorized into uncomplicated and complicated, with the latter now defined as a UTI that encompasses infection beyond the bladder (for instance, pyelonephritis, prostatitis, or UTIs combined with febricity or bacteremia) [2].

For a long time, urine culture has served as the “gold standard” when it comes to identifying these patients. A positive urine culture, traditionally defined as ≥10^5^ colony-forming units (CFU) per milliliter (mL) of a uropathogen, remains the gold standard for diagnosis [3]. Additional supportive criteria may include pyuria (≥10 WBC/mm^3^), direct visualization of organisms on Gram stain, repeated positive urine cultures with ≥10^2^ CFU/mL from non-voided specimens, or ≤10^5^ CFU/mL of a single uropathogen in the context of ongoing effective antimicrobial therapy [4]. These definitions demonstrate the complexity of UTI diagnosis and highlight the limitations of relying on urine culture alone, particularly given its delayed turnaround time and potential discordance with clinical presentation.

However, there are several limits regarding using urine culture as the diagnostic method for UTIs, including long turnaround times, risk of contamination, and sometimes ambiguous correlation with clinical symptoms [1]. These disadvantages highlight the need for a rapid, precise, and standardized way of diagnosing UTI. Under these circumstances, in recent years, advances in automated urinalysis systems have introduced loads of new techniques for not only rapid screening but also semi-quantitative analysis. These analyzers, such as Sysmex UF-5000 [5,6], provide the ability to rapidly quantify the urine sample data and can potentially reduce the reliance on urine culture to make an accurate UTI diagnosis.

Sysmex Corporation (Kobe, Japan) has developed several generations of automated urine particle analyzers using fluorescence flow cytometry technology. The earlier generation included the UF-100 and UF-1000i [7], the latter of which served as the long-standing flagship model widely validated in the literature. The current generation comprises the UN-3000 [8], UF-4000, UF-5000, and UF-1500. Among these, the UF-5000 is the top-of-the-line model and direct successor to the UF-1000i, offering enhanced particle differentiation, a depolarized side scatter channel for improved crystal and red blood cell discrimination, and an integrated body fluid mode. The UF-4000 provides similar fluorescence flow cytometry technology at a slightly reduced feature set [9], while the UF-1500 is a compact variant designed for smaller laboratories with lower throughput requirements [10]. Although these other models exist, published studies rigorously evaluating the diagnostic performance of the UF-4000, UN-3000, and UF-1500 remain limited compared with the extensive literature available for the UF-5000. Because the UF-5000 is the most clinically validated, most widely deployed instrument in its class globally, and the intended successor to the benchmark UF-1000i, we focused our evaluation on this model. This study aims to consolidate and analyze current statistical evidence regarding the accuracy of advanced urine analyzers, mainly the UF-5000, in diagnosing UTIs. Since WBC count and bacterial level are the most commonly used indicators globally, we sorted and synthesized different studies that compared the overall screening or diagnostic performance based on these two metrics.

## 2. Methods

### 2.1. Search Strategy and Eligibility Criteria

**The meta-analysis was conducted based on the Preferred Reporting Items for Systematic Reviews and Meta-analyses (PRISMA) guidelines using the associated flow chart to report the numbers of included and excluded studies at each stage (Supplementary Table S1).** Two independent investigators (K.W. Chang and C.Y. Tsai) conducted a systematic search of PubMed, Embase, Science Direct, and Web of Science from the earliest record from January 2018 to February 2024 with no restrictions on language. Such a timespan was set to ensure that the research fully focused on the performance of UF-5000, which is the latest generation of flow cytometry technology.

We used keywords focusing on the disease (urinary tract infection), the automated analyzing products (UF-5000), and other relevant words (screening, diagnosis, detection). The search was conducted using a combination of MeSH terms and keywords with Boolean operators (AND, OR). The search string for the selected databases was “*(((urinary tract infections[MeSH Terms]) OR (urinary tract infection)) OR (UTI)) AND (UF-5000)*”. We also performed a hand search by manually reviewing the reference lists of the identified articles and relevant clinical guidelines to capture any additional studies that might have been missed by the database search. The reference lists of the included articles and guidelines of the American Urological Association were manually reviewed, and external peer reviewers were asked to identify any additional trials. Of the total of 26 articles that were included via the abovementioned research method, we then examined the studies based on the following inclusion criteria: 1. studies must evaluate the screening or diagnostic performance of UF-5000, 2. the total sample size of the selected studies must be greater than 40 and include more than 10 patients with UTI, and 3. both sensitivity and specificity must be available, and a 2 × 2 table constructable. Several exclusion criteria, on the other hand, were also applied, including the following: 1. duplicate studies, 2. studies conducted in animals, and 3. congress abstracts and letters to the editor. A total of 4 studies were excluded because they did not intend to direct UTI screening. Another 4 studies were not included since they did not include enough information about the performance of the automated screening tools. The remaining 18 studies were used to further conduct the systematic review. No formal protocol was registered for this systematic review.

### 2.2. Data Extraction and Statistical Analysis

Next, research data were independently extracted from the eligible studies by two reviewers (K.W. Chang and C.Y. Tsai). Studies were grouped for synthesis based on the similarity of evaluated parameters and area of focus. For each study, calculated from known data (sample sizes, positive cases, negative cases, sensitivity, and specificity), 2 × 2 tables were constructed based on the four variables: true positives (TP), false negatives (FN), false positives (FP), and true negatives (TN). While 18 studies met the eligibility criteria for the review, only 9 studies provided sufficient raw data (TP, FN, FP, TN) to be partitioned into 2 × 2 tables for the quantitative meta-analysis, with 5 of them focused exclusively on bacterial counts and 4 studies focused on both bacterial and WBC counts separately. For each selected study, the pooled sensitivity and specificity of each group of studies were calculated. Pooled estimates for sensitivity and specificity were calculated using a **bivariate random-effects model**. We then extracted the area under the receiver operating characteristic curve (AUC) and its corresponding standard error (SE), and pooled estimates of AUC with 95% confidence intervals (CIs) were calculated under both fixed-effects and random-effects models. Forest plots were used to visually represent the estimated effect sizes and their corresponding 95% CIs. Analysis of the data was performed using R version 4.5.1 and MedCalc 23.3.7 software. Evidence levels of each study were rated using the leveling system of the Urological Association of Asia and Asian Association of UTI & STI (UAA-AAUS) guidelines published in 2016 [11].

### 2.3. Risk of Bias Assessment

As for quality assessment of the selected studies, we rated them based on the QUADAS-2 score, which evaluates risk of bias across four domains: patient selection, index test, reference standard, and flow and timing. Then, based on the diagnostic indicators used in these articles (WBC count or bacterial count), we classified the studies accordingly. Publication bias was assessed using Deeks’ funnel plot asymmetry test [12]. As for certainty assessment, we used the Grading of Recommendations Assessment, Development and Evaluation (GRADE) framework adapted for diagnostic accuracy studies.

The body of evidence was evaluated through five different domains: risk of bias (results of the QUADAS-2 assessments), inconsistency of results, indirectness of evidence, imprecision of effect estimates, and publication bias. The final certainty was classified into four levels—high, moderate, low, or very low—representing the extent of our confidence that the true diagnostic performance of the UF-5000 is close to the estimated effect.

## 3. Results

**Figure 1:**
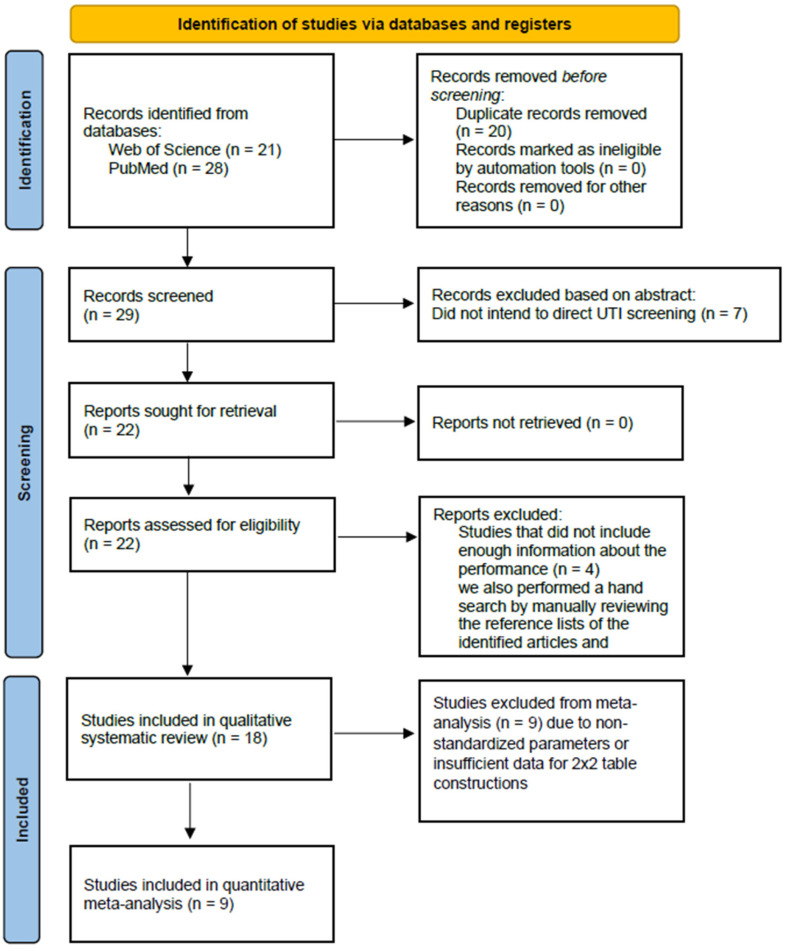
Flow chart of the article selection process.

In a total of 18 studies [5,6,13,14,15,16,17,18,19,20,21,22,23,24,25,26,27,28], 25,337 total samples were included, with the samples collected from UTI-suspected patients in the outpatient clinic, the emergency department, and hospitalized patients. **The literature search and study selection process are summarized in the PRISMA flow diagram (**Figure 1**).** All of the studies used UF-5000 as the primary tool to screen for UTIs. The evidence hierarchy of the studies, rated based on the AAUS guidelines [11], was level 3. This classification mainly reflected the observational nature of the available data, as the majority of studies were prospective or retrospective diagnostic accuracy studies without randomization or intervention-based comparisons. Most investigations evaluated the performance of UF-5000 in routine clinical settings, with urine culture being used as the reference standard. Although none of the included studies were randomized controlled trials, the consistent use of standardized diagnostic reference criteria and the inclusion of real-world patient populations supported the applicability of these findings to daily clinical practice.

The characteristics of the included studies and their primary findings are summarized in Table 1. Out of the 18 selected studies, nine studies [13,15,16,17,19,24,26,27,28] included methods that perform UTI screening based on bacterial counts, and four studies [17,19,24,28] used WBC counts. Data were extracted from these studies and calculated. Data from the rest of the studies [5,6,14,18,20,21,22,23,25], either using combined data or other items (such as Gram stain results, yeast-like cells, or urine conductivity), could not be constructed into 2 × 2 tables and were therefore excluded. 

**Figure 2:**
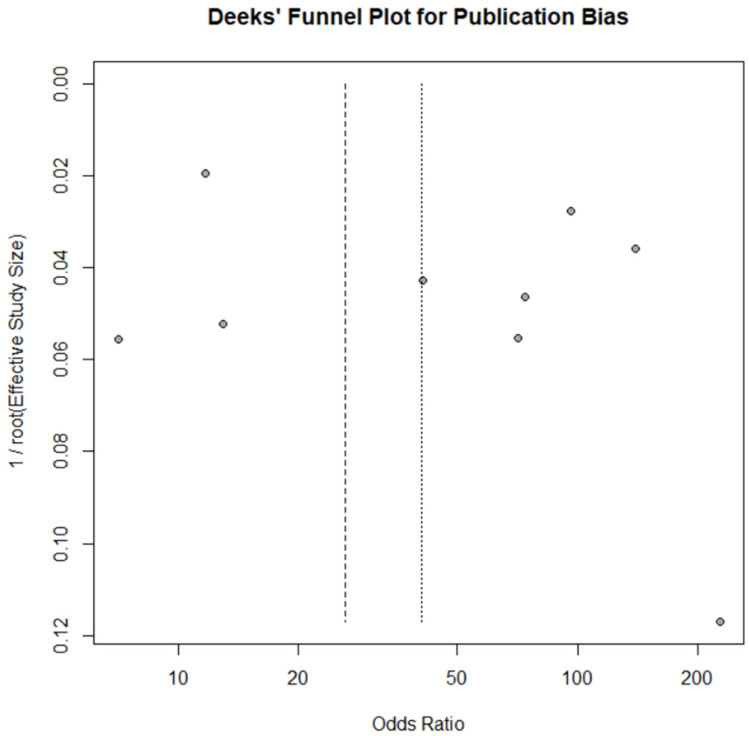
Deeks’ funnel plot for publication bias.

Furthermore, Table 2 presents a summary of the QUADAS-2 scoring of the included studies. Overall, most studies had a low to moderate risk of bias. As the majority of the studies were designed similarly, the main concern lies in post hoc threshold determination and the lack of explicit reporting on blinding. Publication bias assessment results using Deeks’ funnel plot asymmetry test were illustrated in Figure 2.

### 3.1. Bacterial Count and UTI

**Figure 3:**
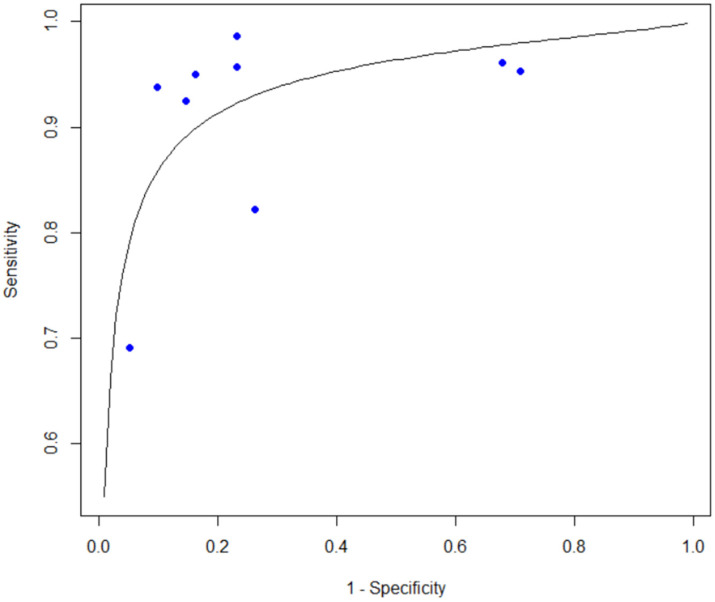
SROC curve of the included bacterial count studies.

For the bacteria detection group containing nine studies, the pooled sensitivity was 0.927 (95% confidence interval [CI], 0.872–0.959), pooled specificity was 0.751 (95% CI, 0.558–0.878), pooled positive likelihood ratio (PLR) was 3.72 (95% CI, 2.10–7.64), and pooled negative likelihood ratio (NLR) was 0.10 (95% CI, 0.05–0.23). The resulting diagnostic odds ratio (DOR) was 38.4 (95% CI, 9.2–162.9). The summary ROC analysis demonstrated an area under the curve of 0.932 (Figure 3).

### 3.2. WBC Count and UTI

**Figure 4:**
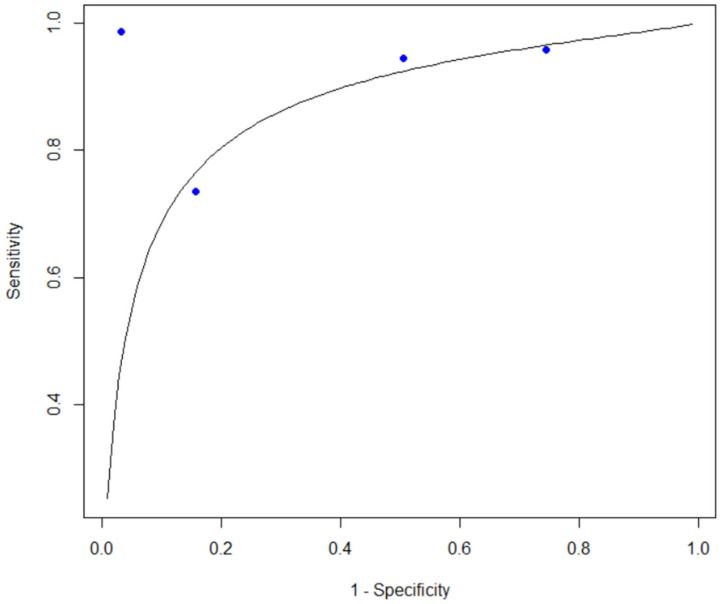
SROC curve of the included WBC count studies.

For the WBC detection group containing four studies, the pooled sensitivity was 0.897 (95% CI, 0.755–0.961), and the pooled specificity was 0.600 (95% CI, 0.293–0.844). PLR was 2.24 (95% CI, 1.27–5.75), whereas NLR was 0.17 (95% CI, 0.05–0.47). The resulting DOR was 13.2 (95% CI, 2.7–64.0). Summary ROC analysis yielded an area under the curve (AUC) of 0.867 (Figure 4).

### 3.3. Discrimination of Gram-Positive vs. Gram-Negative Infections

We also performed a pooled analysis of the ability of UF-5000 to discriminate between Gram-positive infection, Gram-negative infection, or mixed flora. Out of the 18 selected studies, four studies [6,14,15,19] were included in this pooled study. The kappa value of the selected studies ranged from 0.26 (95% CI, 0.15–0.36) to 0.88 (95% CI, 0.83–0.91). The random-effects meta-analysis yielded a pooled kappa of 0.52 (95% CI, 0.08–0.79), indicating a moderate level of agreement overall. However, there was substantial heterogeneity across studies (Q = 133.0, *p* < 0.001; I^2^ = 98.8%), suggesting considerable variability in agreement estimates depending on study settings and populations.

### 3.4. Publication Bias

Publication bias was assessed using the Deeks’ funnel plot asymmetry test. However, analysis was performed only for the bacterial count subgroup due to the limited number of studies in other subgroups. The assessment of the bacterial count subgroup showed no significant asymmetry (*p* = 0.369). Visual inspection of the Deeks’ funnel plot did not reveal obvious asymmetry, suggesting no apparent small-study effects. Although Deeks’ asymmetry test did not reveal statistically significant publication bias, given that there were fewer than 10 studies included, the results should be interpreted with caution.

### 3.5. Certainty Assessment

Based on the GRADE assessment, the overall certainty of evidence for the diagnostic accuracy of Sysmex UF-5000 was rated as moderate. While the majority of included studies exhibited a low risk of bias and provided direct evidence within the target clinical populations, the certainty was downgraded by one level due to significant inconsistency. This heterogeneity mainly stemmed from substantial variations in optimal bacterial (BACT) and white blood cell (WBC) cut-off values across different clinical settings and the inconsistent performance of bacterial flags in discriminating Gram-positive pathogens. However, the certainty for the UF-5000’s high negative predictive value (NPV) remained robust across most studies, supporting its reliability as a rule-out tool for urinary tract infections. No serious concerns were identified regarding indirectness or imprecision for the primary screening outcomes, and we did not discover publication bias when reviewing the included studies.

## 4. Discussion

This systematic review and meta-analysis demonstrate that the Sysmex UF-5000 urine flow cytometry system provides reliable screening efficacy for urinary tract infections, with consistently high pooled sensitivity and low negative likelihood ratios across studies. These findings support the role of UF-5000 as a clinically useful screening tool for suspected UTIs in diverse clinical settings, including outpatient clinics, emergency departments, and inpatient wards.

### 4.1. Clinical Role of UF-5000 as a Rule-Out Screening Tool

Among the analyzed parameters, bacterial count measured by UF-5000 showed a pooled sensitivity of 0.927 and a negative likelihood ratio of approximately 0.10, indicating fair power for screening purposes, which supports the device’s utility as a rule-out tool. The primary unmet need in UTI diagnostics is not confirmation—traditionally achieved by urine culture—but rather the rapid exclusion of infection to reduce unnecessary cultures and empiric antibiotic use [4]. In this regard, the UF-5000 aligns well with antimicrobial stewardship principles by enabling clinicians to safely defer urine culture and antibiotic initiation in low-risk patients with negative screening results.

Although pooled specificity was moderate (0.751 for bacterial count and 0.600 for WBC count), this trade-off is acceptable for a screening modality designed to minimize false-negative results. Importantly, the diagnostic odds ratios observed in both bacterial and WBC-based analyses further reinforce the overall discriminatory power of UF-5000, supporting its implementation as an initial screening tool rather than a definitive diagnostic substitute for urinary culture.

### 4.2. Relationship to Current Guideline Frameworks

Despite the favorable diagnostic performance observed in this meta-analysis, current clinical guidelines from organizations, such as the IDSA and ASM, continue to emphasize urine culture results in the definition of UTI [29]. This discrepancy does not reflect a lack of technological capability but rather a lack of outcome-driven evidence linking automated urinalysis to patient-centered endpoints, such as symptom resolution, antibiotic exposure, or clinical complications. Our findings provide robust diagnostic accuracy evidence but do not directly address clinical outcomes, which remain a critical barrier to guideline integration. Future guideline updates may reasonably consider UF-5000 as a recommended screening modality once standardized thresholds and prospective outcome data become available.

### 4.3. Comparison with Previous-Generation Urine Flow Cytometry Systems

Compared with earlier-generation analyzers such as the UF-1000i, the UF-5000 demonstrates modest but meaningful improvements in diagnostic performance, particularly in specificity and overall discriminatory ability. Prior meta-analyses of UF-100/UF-1000i systems reported pooled sensitivities ranging from 0.87 to 0.92 and specificities between 0.60 and 0.67 [7]. In contrast, the UF-5000 showed improved pooled specificity for bacterial detection (0.751) while maintaining high sensitivity, likely reflecting technological advancements such as blue-laser fluorescence, nucleic acid staining, and refined signal-processing algorithms. Although direct head-to-head comparisons were not available, these findings suggest that UF-5000 may mitigate some limitations of earlier systems, particularly false-positive results related to non-bacterial particles.

### 4.4. Gram-Negative Versus Gram-Positive Discrimination

An additional feature of the UF-5000 is its ability to provide early Gram classification [27]. Our pooled analysis demonstrated a moderate overall agreement (pooled kappa 0.52) between UF-5000 Gram flags and conventional culture results, with substantial heterogeneity across studies. Notably, performance was consistently better for Gram-negative organisms, which account for the majority of UTIs, including the Escherichia coli and Klebsiella species. Early identification of Gram-negative dominance may therefore assist in preliminary risk stratification and empirical decision-making while awaiting culture confirmation.

However, the high degree of heterogeneity and reduced agreement in Gram-positive or mixed-flora samples underscore important limitations. Overlapping fluorescence characteristics, lower bacterial loads, and polymicrobial interference likely contribute to reduced accuracy in these contexts. As a result, Gram-positive or mixed-flora flags generated by UF-5000 should be interpreted cautiously and should not be used as the only basis for antimicrobial selection. Gram classification by UF-5000 should be regarded as a supplementary tool rather than a replacement for culture-based identification.

### 4.5. Heterogeneity and Real-World Applicability

Substantial heterogeneity was observed across most pooled analyses, which is expected in diagnostic meta-analyses encompassing diverse clinical environments (Table 1). The primary source of inconsistency is the wide variation in diagnostic thresholds (cut-off values) across studies, which ranged significantly for both bacterial and WBC counts. Importantly, many studies derived optimal thresholds post hoc using ROC analysis rather than applying predefined cut-offs, potentially inflating diagnostic performance estimates. Furthermore, the lack of a standardized reference criterion for “positive culture”—with definitions varying from 10^3^ to 10^5^ CFU/mL—also introduced substantial variability across the analysis. Additionally, variations in patient populations, clinical settings, UTI prevalence, urine collection methods, and sample handling all contribute to between-study variability.

### 4.6. Contamination and Diagnostic Gray Zones

A persistent challenge in UTI diagnostics is distinguishing true infection from contamination or asymptomatic bacteriuria. UF-5000, like other automated systems, cannot reliably differentiate contamination from polymicrobial infection, particularly in voided urine samples [30]. Mixed-flora flags and low-level bacterial counts should therefore prompt careful clinical correlation rather than immediate diagnostic conclusions. Integration of UF-5000 results with clinical symptoms, collection methods, and repeat testing strategies remains essential to avoid overdiagnosis.

### 4.7. Limitations

Several limitations of this meta-analysis should be highlighted. First, the most significant drawback of the UF-5000 is its inability to provide pathogen identification and antimicrobial susceptibility testing (AST), which is essential for managing infections, especially in this era of widespread antimicrobial resistance. While UF-5000 has the potential to serve as a reliable screening tool, the diagnostic gap between flow cytometry screening and the traditional urinary culture remains significant. Second, as mentioned previously in Section 4.5, high statistical heterogeneity across the selected studies was observed, which likely stems from variations in clinical settings, patient populations, and the lack of standardized cut-off values for bacterial and WBC counts. Such shortcomings limit the replicability of the studies. Finally, most selected studies determined their optimal cut-off values post hoc using ROC curve analysis. Such research methods can lead to an overestimation of diagnostic performance compared to using predefined clinical thresholds in real-world practice.

### 4.8. Future Directions

Several areas warrant further investigation. First, prospective studies validating standardized bacterial and WBC cut-offs are essential to improve reproducibility and clinical adoption. Second, outcome-based studies evaluating the impact of UF-5000–guided screening on antibiotic utilization, culture reduction rates, emergency department length of stay, and patient outcomes are critically needed. Third, algorithm-based approaches integrating UF-5000 data with dipstick results and clinical presentation may enhance diagnostic precision. Advanced computational methods, such as machine learning, may come in handy in identifying complex patterns within flow cytometry data [31,32]. Incorporating such tools into routine practice could potentially reduce the rate of false positives and further promote the clinical utility of automated urinalysis platforms. Finally, targeted studies in special populations—such as elderly patients, catheterized individuals, and patients with neurogenic bladder—are necessary to define population-specific performance characteristics.

## 5. Conclusions

Overall, this meta-analysis supports the UF-5000 as a rapid and clinically valuable screening instrument for suspected UTIs. Its high sensitivity and rule-out capability make it particularly suitable for frontline use in routine practice, while its Gram-negative discrimination offers limited but meaningful adjunctive information. Although urine culture remains indispensable for definitive diagnosis and antimicrobial susceptibility testing, UF-5000 represents a significant step forward in modernizing UTI diagnostics and supporting antimicrobial stewardship.

## Figures and Tables

**Table 1:** Summary of the selected studies.

Year	Country	Author	Reference Standard	Cut-Off Value	Total Samples	With UTI	Without UTI	Contaminated	TP	FP	TN	FN
2024	Denmark ([28])	Hertz	Culture, >10^3^ CFU/mL or >10^4^ CFU/mL (two different labs)	BACT > 7/µL	512	213	299	NA	203	212	87	10
				WBC > 3.2/µL	512	213	299	NA	204	223	76	9
2023	Brazil ([13])	Toledo	Culture, >10^4^ CFU/mL (pure culture), >10^5^ CFU/mL (two different colonies)	BACT ≥ 100/μL	3569	761	2570	238	960	1743	827	39
2023	China ([15])	Wang	Culture, >10^4^ CFU/mL	BACT ≥ 1367/μL	671	246	425	NA	170	22	403	76
2023	China ([14])	Chen	Culture, >10^4^ CFU/mL	WBC ≥ 32.20/μL (male) and ≥ 39.15/μL (female) and BACT ≥ 22.35/μL (male) and ≥127.25/μL (female)	2600	425	2175	9	unknown	unknown	unknown	unknown
2022	Poland ([18])	Szmulik	Culture, >10^5^ CFU/mL	WBC ≥ 40/µL or BACT ≥ 300/µ	106	32	41	33	32	7	34	0
2022	Republic of Korea ([16])	Kim	Culture, >10^5^ CFU/mL	BACT ≥ 685.3/µL	1063	177	886	NA	168	216	670	9
2022	Indonesia ([19])	Christy	Culture, >10^5^ CFU/mL	WBC > 87.2/μL	100	70	30	NA	69	1	29	1
				BACT > 582.22/μL	100	70	30	NA	69	7	23	1
2022	China ([17])	Chun	Culture, >10^5^ CFU/mL	BACT ≥ 196 units/μL	383	90	293	NA	74	77	216	16
				WBC ≥ 14.7 cells/μL	383	90	293	NA	85	148	145	5
2022	Switzerland ([20])	Müller	Culture, >10^4^ CFU/mL	WBC < 25/µL, BACT < 125/µL, SEC < 31/µL = No significant urine culture growth	3835	817	2501	517	154	82	669	54
2021	Taiwan ([6])	Yang	Culture, >10^3^ CFU/mL	Gram negative	102	102	Excluded	24	52	4	30	13
2021	Denmark ([22])	Alenkaer	Culture, >10^8^ CFB/L	BACT count ≥ 50x106/L or YLC ≥ 25 × 10^6^/L	1119	494	625	NA	492	387	238	2
2021	Norway ([23])	Gilboe	Culture, > 10^4^ CFU/mL (primary pathogen), >10^5^ CFU/mL (secondary pathogen)	BACT count ≥ 100.000/mL and WBCs ≥ 10/μL,	1000	223	777	NA	196	383	394	27
2021	Norway ([21])	Haugum	Culture, >10^4^ CFU/mL	BACT ≥ 30/μL, WBC ≥ 30/μL	3919	1998	1470	937	1930	654	816	68
2020	Austria ([24])	Enko	Culture, >10^5^ CFU/mL	BACT ≥ 135/µL	344	98	246	NA	98	35	203	8
				WBC ≥ 23/µL	344	98	246	NA	72	39	207	26
2020	Italy ([25])	Ippoliti	Culture, >10^3^ CFU/mL	Negative if SEC ≥ 30/µL, or urine conductivity < 6 mS/cm, or WBC < 5/µL	1295	248	988	59	179	69	1047	0
2018	Italy ([5])	De Rosa	Culture, >10^5^ CFU/mL	BACT ≥ 58/μL and/or YLC ≥ 150/μL	2719	1922	797	152	792	419	1503	5
2018	Republic of Korea ([27])	Kim	Culture, >10^3^ CFU/mL	BACT ≥ 71/μL	1430	336	1094	NA	319	179	915	17
2018	China ([26])	Ren	Culture, >10^4^ CFU/mL	BACT > 30/μL	498	93	405	NA	89	94	311	4

**Table 2:** Summary of the QUADAS-2 scoring of the selected studies.

Country, Year	Patient Selection	Index Test (UF-5000)	Reference Standard	Flow & Timing	Overall Risk
Denmark, 2024 ([28])	Low	Unclear	Low	Low	Low to Moderate
Brazil, 2023 ([13])	Low	Unclear	Low	Low	Low to Moderate
China, 2023 ([14])	Low	Unclear	Low	Low	Low to Moderate
China, 2023 ([15])	Low	Unclear	Low	Low	Low to Moderate
Republic of Korea, 2022 ([16])	Low	Unclear	Low	Low	Low to Moderate
China, 2022 ([17])	Low	Unclear	Low	Low	Low to Moderate
Poland, 2022 ([18])	Low	Unclear	Low	Low	Low to Moderate
Indonesia, 2022 ([19])	High	Low	Low	Low	Moderate to High
Switzerland, 2022 ([20])	Low	Unclear	Low	Low	Low to Moderate
Norway, 2021 ([21])	Low	Unclear	Low	Low	Low to Moderate
Denmark, 2021 ([22])	Low	Unclear	Low	Low	Low to Moderate
Norway, 2021 ([23])	Low	Unclear	Low	Low	Low to Moderate
Taiwan, 2021 ([6])	Low	Unclear	Low	Low	Low to Moderate
Austria, 2020 ([24])	Low	Low	Low	Low	Low
Italy, 2020 ([5])	High	Low	Low	Low	Moderate to High
China, 2018 ([26])	Low	Unclear	Low	Low	Low to Moderate
Italy, 2018 ([25])	Low	Unclear	Low	Low	Low
Republic of Korea, 2018 ([27])	Low	Unclear	Low	Low	Low to Moderate

## Data Availability

All data generated or analyzed during this study are included in this published article and its additional file.

## Supplementary Materials

The following supporting information can be downloaded at: https://www.mdpi.com/article/10.3390/diagnostics16091275/s1, Table S1: PRISMA Checklist.

## Abbreviations

The following abbreviations are used in this manuscript:

LUTS	lower urinary tract symptoms
UDS	urodynamic studies
